# Differential Effects of Selenium and Trehalose on Growth, Nutritional Quality, and Antioxidant Properties of Mustard Sprouts

**DOI:** 10.3390/plants15213371

**Published:** 2026-11-01

**Authors:** Shuyu Han, Renjing Feng, Yifeng Gu, Jie Ma, Xuena Yu, Huanhuan Huang, Yi Tang, Huanxiu Li, Fen Zhang, Bo Sun, Zhi Huang

**Affiliations:** 1 College of Horticulture, Sichuan Agricultural University, Chengdu 611130, China; 2024305095@stu.sicau.edu.cn (S.H.);; 2 Bijie Institute of Agricultural Science, Bijie 551700, China

**Keywords:** mustard sprouts, selenium, trehalose, growth, nutritional quality, antioxidant properties

## Abstract

Mustard sprouts are rich in nutrients and have considerable potential as functional vegetables. In this study, we evaluated the effects of selenium, trehalose, and their combination on the growth and nutritional quality of mustard sprouts. Selenium and trehalose treatments reportedly enhanced the nutritional attributes of the sprouts. Selenium alone had the strongest growth-promoting effect, increasing edible fresh weight and plant height to 1.09- and 1.10-fold of the control, respectively. Both individual and combined treatments of selenium and trehalose enhanced nutrient accumulation and antioxidant capacity. Notably, selenium treatment alone increased the soluble sugar, soluble protein, ascorbic acid, flavonoid, and total phenolic contents to 1.09-, 1.23-, 1.06-, 1.29-, and 1.62-fold of the control, respectively. Trehalose alone and the combined selenium–trehalose treatment markedly increased total indolic glucosinolates accumulation, whereas selenium alone significantly reduced its content, and the combined treatment was especially advantageous in the accumulation of indolic glucosinolates. Collectively, selenium treatment alone effectively improves the nutritional quality and antioxidant capacity of mustard sprouts, while the application of Tre alone and the combined treatment promoted the accumulation of secondary metabolites, accompanied by the inhibition of plant growth and the decrease in photosynthetic pigment content, providing a basis for regulating their nutritional quality.

## 1. Introduction

Mustard (*Brassica juncea*) is a widely cultivated edible vegetable in the *Brassicaceae* family. It contains abundant bioactive compounds, including ascorbic acid, phenolic compounds, and glucosinolates (GS), and GS together with their degradation products exhibit potent antitumor activities [1,2]. Mustard sprouts are young seedlings harvested shortly after germination and are characterized by high nutritional value and a crisp texture. They also offer several production advantages, including a short cultivation period, low input costs, and a pollution-free production system [3,4]. Compared with mature vegetables, sprouts generally contain higher levels of bioactive compounds, contributing to their popularity among consumers [1].

As an essential trace element for humans, selenium (Se) influences plant growth and development while supporting human physiological health [5]. Preharvest Se application improves the postharvest quality of purple-leaf mustard by enhancing antioxidant enzyme activity and modulating anthocyanin, volatile, and lipid metabolism [6]. Optimal Se supply promotes the growth and yield of radish sprouts, increases soluble sugar, protein, and antioxidant contents, and upregulates antioxidant biosynthetic genes. It also enhances the antioxidant enzyme activities while increasing endogenous Se and anthocyanin accumulation [7,8]. In broccoli sprouts, Se increases anthocyanin, flavonoid, and glucosinolate concentrations and enhances antioxidant capacity and stress resistance [9,10]. In addition, studies have found that *Cruciferae* sprouts can be biofortified by Se to accumulate Se-methylselenocysteine (SeMSCys) without negatively affecting the chemically preventive glucosinolate content [11]. Some studies have also pointed out that Se biofortification can significantly increase the total phenolic content of *Cruciferous* plants in general, and the regulation of Se on secondary metabolism is affected by many factors [12]. At the same time, the uptake and assimilation of Se in *Cruciferous* plants are closely dependent on the sulfur metabolic pathway. Se can be integrated into the glucosinolate skeleton to form selenoglucosinolate, which provides a biochemical basis for the interaction between Se and glucosinolate metabolism [13]. Therefore, elucidating how Se regulates plant development and nutrient accumulation is of considerable importance.

Trehalose (Tre) is a unique non-reducing disaccharide composed of two glucose units. Although present at very low levels in plants, Tre can accumulate substantially under stress conditions and enhance tolerance to various abiotic stresses. As an osmoprotectant, Tre maintains cellular osmotic homeostasis and suppresses reactive oxygen species (ROS) accumulation. Studies have shown that Tre improves cold tolerance in melon seedlings, enhances grape adaptability to low temperature, salinity, and drought, and helps maintain normal plant growth and metabolism [14,15]. In addition, exogenous Tre alleviates sodium chloride (NaCl) stress-induced damage in Indian mustard by maintaining ion homeostasis, preserving cell membrane integrity, and activating the antioxidant system [16]. It can also regulate carbohydrate and secondary metabolic pathways to enhance heat tolerance in *Ginkgo biloba* leaves [17]. Most existing studies on Tre have focused on its role in stress tolerance. However, recent evidence indicates that Tre also regulates plant growth and quality under non-stress conditions. For example, exogenous Tre can improve photosynthetic performance, root activity, and nutrient uptake efficiency, thereby increasing the yield and quality of Indian mustard [18]. It can also reshape metabolic profiles in mustard sprouts and promote the accumulation of soluble sugar, ascorbic acid, and total phenolic compounds [19]. In addition, Tre has high thermal stability, is resistant to hydrolysis, is inexpensive, and has low toxicity, making it a promising exogenous plant regulator for agricultural production [20]. Nevertheless, the mechanisms underlying Tre-mediated regulation of crop quality and its application potential under non-stress conditions still need to be further explored.

Se and Tre, as exogenous regulatory substances, can improve plant growth, nutrient accumulation, and antioxidant capacity, but their modes of action differ. Most of the existing studies have examined the effects of Se or Tre individually in different plant species or varieties, and the reported responses vary considerably. Whether their combined application produces synergistic or antagonistic effects and, consequently, different effects on plant growth and quality formation remains still requires further investigation. Moreover, comparative studies of single and combined Se and Tre treatments in the mustard variety ‘Chuanbao 18’, particularly their differential effects on sprout growth, nutritional composition, and antioxidant properties, remain to be undertaken. Therefore, this study systematically investigated the effects of Se, Tre, and their combined application on the growth and quality of ‘Chuanbao 18’ mustard sprouts and identified the optimal treatment for improving sprout quality. The findings provide a theoretical basis for high-quality cultivation and precise quality regulation of mustard sprouts.

## 2. Results

### 2.1. Growth of Sprouts and Se Content

**Figure 1:**
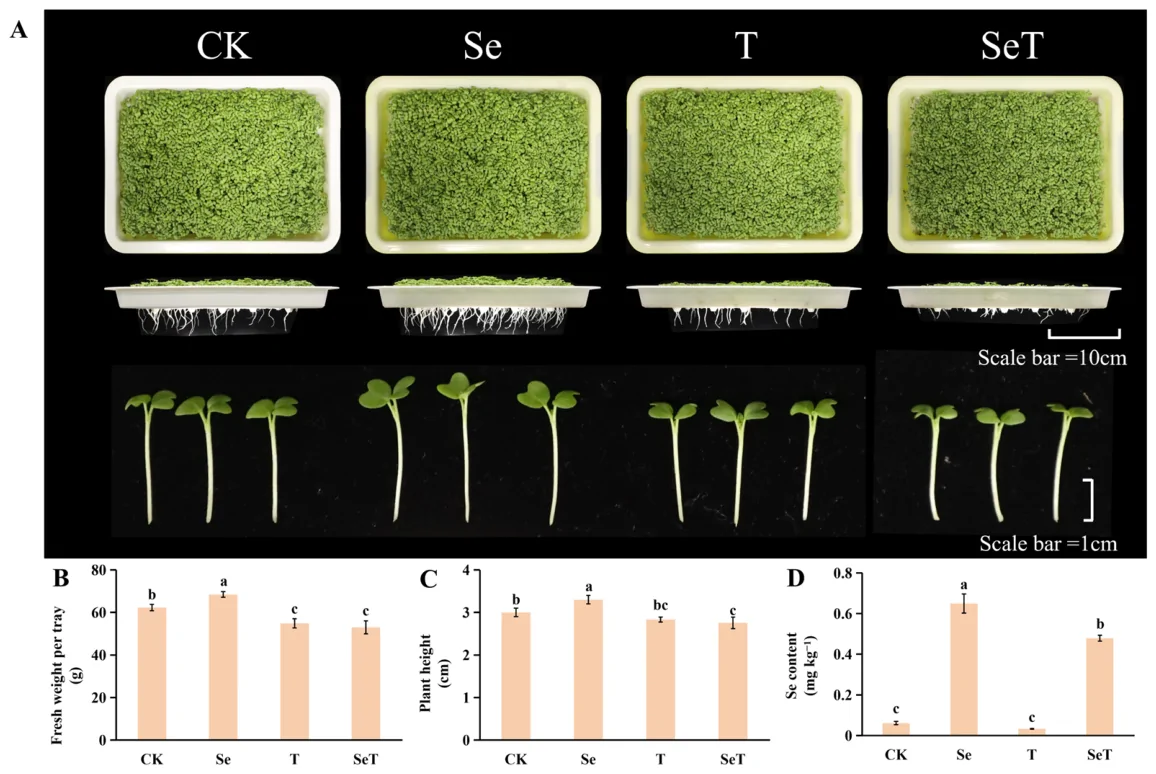
Growth and related physiological parameters of mustard sprouts under different treatments. (**A**) Distilled water (CK), sodium selenite (Se), trehalose (T), and sodium selenite + trehalose (SeT); (**B**) fresh weight per tray; (**C**) plant height; (**D**) Se content. Different letters indicate statistically significant differences among treatments (*p* < 0.05). Two-way ANOVA results for main effects and interaction effects are detailed in Supplementary Table S1.

Mustard sprout growth was enhanced by Se treatment but inhibited by T and SeT treatments (Figure 1A). Compared with CK, Se treatment significantly increased fresh weight and plant height by 1.09- and 1.10-fold, respectively (Figure 1B,C). However, in the presence of T, the growth-promoting effect of Se disappeared, and the fresh weight and plant height of SeT treatment were not significantly different from those of T treatment being significantly lower than those of Se treatment, indicating that T treatment changed the effect of Se treatment on growth. Se treatment also markedly enhanced Se accumulation in the sprouts. Se content under Se treatment was 10.54- and 7.78-fold higher than in the CK and T treatments, respectively. Se content under SeT treatment was 19.68- and 14.52-fold higher than that of the CK and T treatments, respectively, but significantly lower than that of Se treatment, indicating that the presence of T weakened the cumulative effect of Se treatment on Se content (Figure 1D).

### 2.2. Chlorophylls and Carotenoids

**Figure 2:**
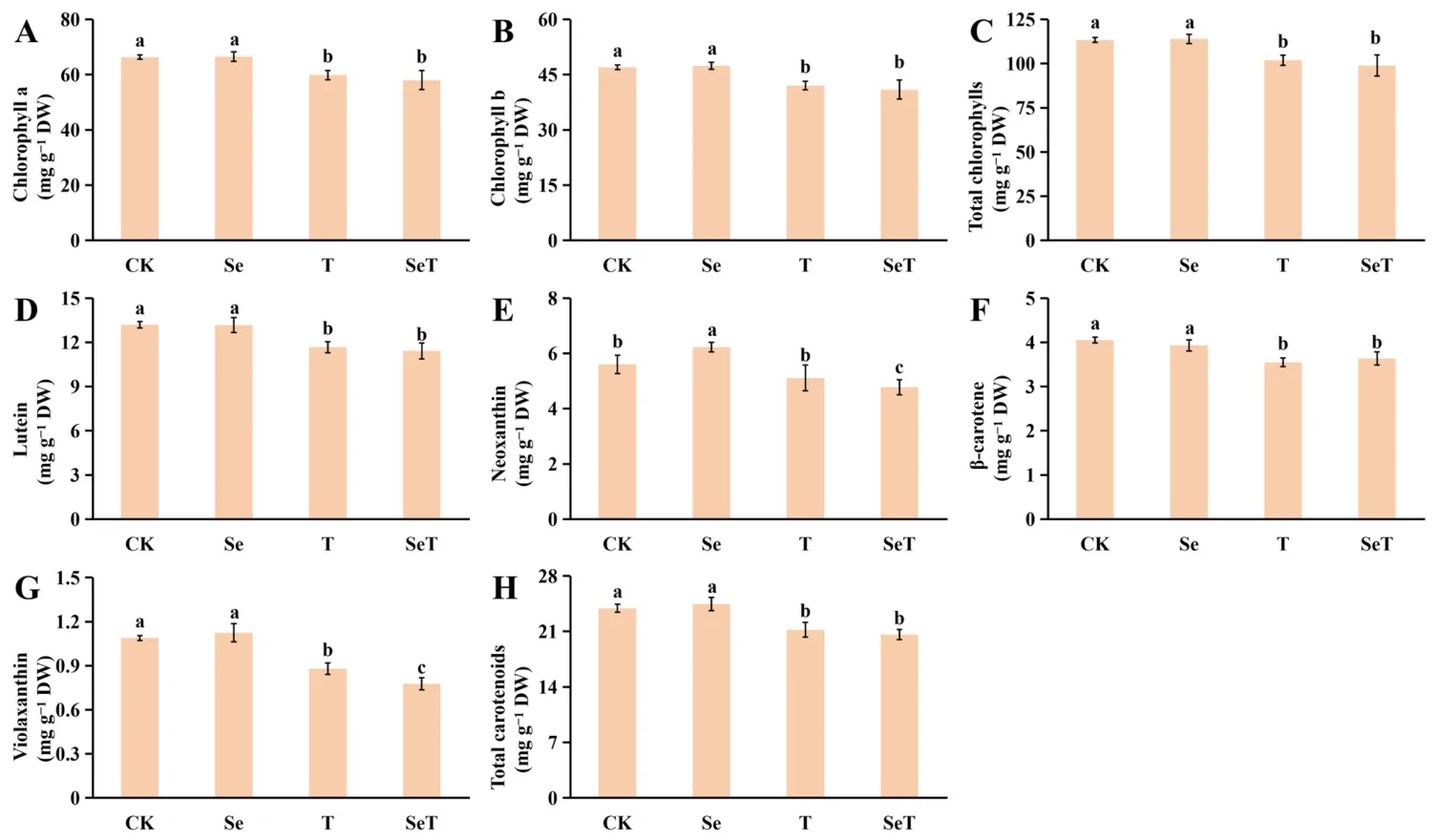
Chlorophyll and carotenoid content of mustard sprouts under different treatments. (**A**) Chlorophyll a; (**B**) chlorophyll b; (**C**) total chlorophylls; (**D**) lutein; (**E**) neoxanthin; (**F**) β-carotene; (**G**) violaxanthin; (**H**) total carotenoids. Different letters indicate statistically significant differences among treatments (*p* < 0.05). Two-way ANOVA results for main effects and interaction effects are detailed in Supplementary Table S1.

Two chlorophylls and four carotenoids were detected in mustard sprouts (Figure 2A–H). Compared with CK, Se treatment did not significantly affect the content of chlorophyll a, chlorophyll b, or total chlorophylls, whereas T and SeT treatments significantly reduced all three, indicating that the decrease in chlorophyll content was mainly caused by T treatment, and the presence of Se did not change the inhibitory effect of T treatment (Figure 2A–C). Among the four carotenoids, lutein was the most abundant, followed by neoxanthin and β-carotene, whereas violaxanthin was the least abundant (Figure 2D–G). Lutein, β-carotene, and total carotenoids content showed similar responses: they were unaffected by Se treatment but significantly decreased under T and SeT treatments (Figure 2D,F,H). Compared with CK, Se treatment significantly increased neoxanthin content, while SeT treatment significantly decreased it (Figure 2E). Violaxanthin content was not significantly affected by Se treatment but decreased sharply under T and SeT treatments, with a greater reduction under SeT treatment (Figure 2G). This indicated that the decrease of carotenoid content was mainly caused by T treatment, and the presence of Se did not change the inhibitory effect of T treatment.

### 2.3. Nutritional Components and Antioxidants

**Figure 3:**
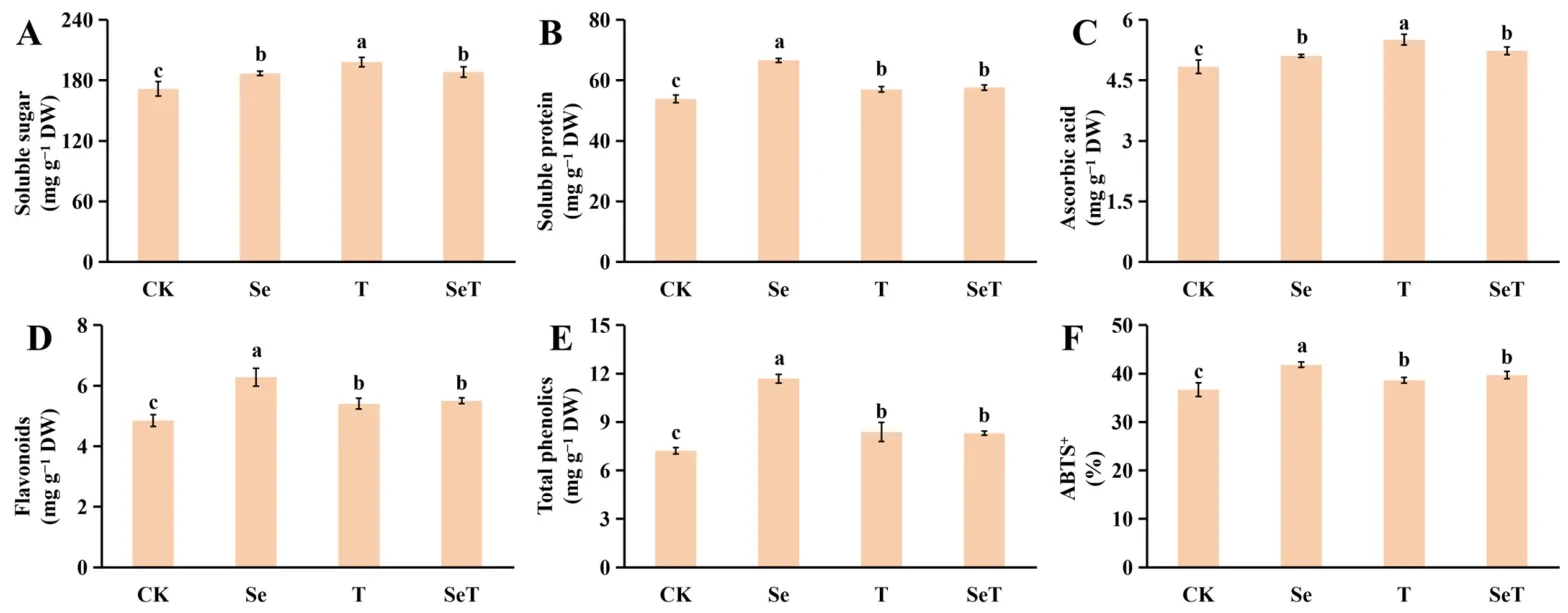
Major nutritional components and antioxidants of mustard sprouts under different treatments. (**A**) Soluble sugar; (**B**) soluble protein; (**C**) ascorbic acid; (**D**) flavonoids; (**E**) total phenolics; (**F**) ABTS^+^. Different letters indicate statistically significant differences among treatments (*p* < 0.05). Two-way ANOVA results for main effects and interaction effects are detailed in Supplementary Table S1.

Soluble sugar and protein content were significantly increased by Se, T, and SeT treatments. Soluble sugar content reached 1.09-, 1.15-, and 1.10-fold those of CK, whereas soluble protein content reached 1.23-, 1.06-, and 1.07-fold those of CK under Se, T, and SeT treatments, respectively (Figure 3A,B). It is worth noting that the soluble protein content under Se treatment was significantly higher than that under T and SeT treatments, indicating that the presence of T weakened the promotion effect of Se treatment on soluble protein. All three treatments also enhanced antioxidant capacity relative to CK. Under Se treatment, ascorbic acid, flavonoid, and total phenolic contents increased to 1.06-, 1.29-, and 1.62-fold those of CK, respectively; under T treatment, they reached 1.14-, 1.16-, and 1.16-fold, respectively, and under SeT treatment, 1.08-, 1.13-, and 1.15-fold, respectively. Among the treatments, Se exerted the strongest stimulatory effect. In addition, Se, T, and SeT treatments all significantly increased 2,2-azinobis(3-ethyl-benzothiazoline-6-sulfonic acid) (ABTS^+^) radical-scavenging capacity compared with CK (Figure 3C–F). It indicated that the presence of T weakened the promoting effect of Se treatment on antioxidant capacity.

### 2.4. Glucosinolates

**Figure 4:**
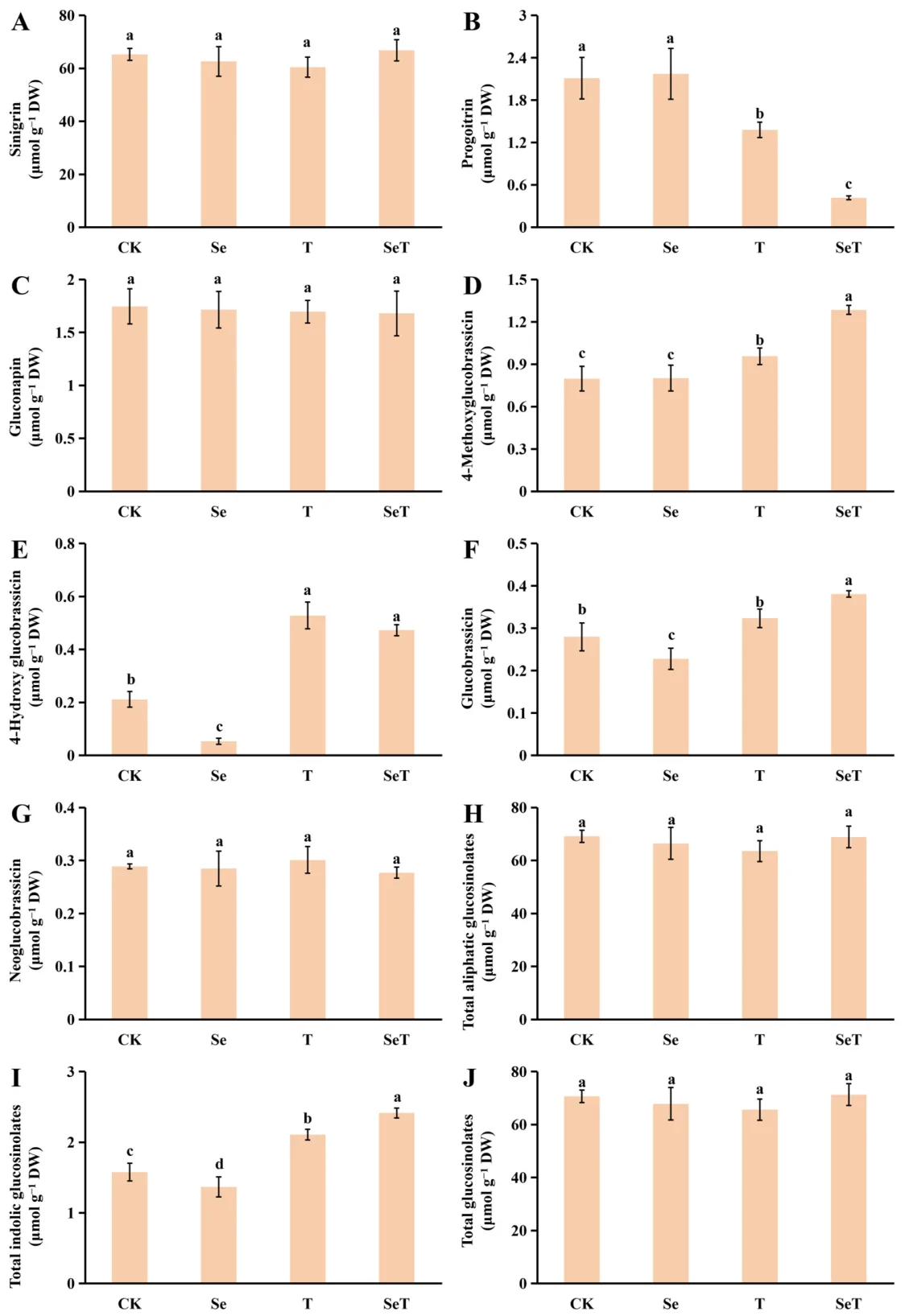
Glucosinolate content of mustard sprouts under different treatments. (**A**) Sinigrin; (**B**) gluconapin; (**C**) progoitrin; (**D**) 4-methoxyglucobrassicin; (**E**) 4-hydroxyglucobrassicin; (**F**) neoglucobrassicin; (**G**) glucobrassicin; (**H**) total aliphatic glucosinolates; (**I**) total indolic glucosinolates; (**J**) total glucosinolates. Different letters indicate statistically significant differences among treatments (*p* < 0.05). Two-way ANOVA results for main effects and interaction effects are detailed in Supplementary Table S1.

Three aliphatic and four indolic glucosinolates were detected in mustard sprouts. The aliphatic glucosinolates comprised sinigrin, gluconapin, and progoitrin, whereas the indolic glucosinolates comprised 4-methoxyglucobrassicin, 4-hydroxyglucobrassicin, neoglucobrassicin, and glucobrassicin. Sinigrin and gluconapin content were not significantly affected by any treatment relative to CK, whereas progoitrin content was significantly reduced by T and SeT treatments and further decreased in the SeT treatment, indicating that the inhibitory effect of T treatment on progoitrin was stronger in the presence of Se. Compared with CK, the T and SeT treatments significantly increased the content of 4-methoxyglucobrassicin, 4-hydroxyglucobrassicin, glucobrassicin, and total indolic glucosinolates. Among them, SeT treatment further increased the content of 4-methoxyglucobrassicin, glucobrassicin, and total indolic glucosinolates. In contrast, Se treatment significantly decreased the content of 4-hydroxyglucobrassicin, glucobrassicin, and total indolic glucosinolates. This indicated that T treatment can increase indolic glucosinolate content, and the presence of Se further enhances the promotion effect of T treatment so that the content under SeT treatment reaches the highest level. Neoglucobrassicin, total aliphatic glucosinolates, and total glucosinolate contents did not differ significantly among treatments (Figure 4A–J).

### 2.5. Principal Component Analysis (PCA)

**Figure 5:**
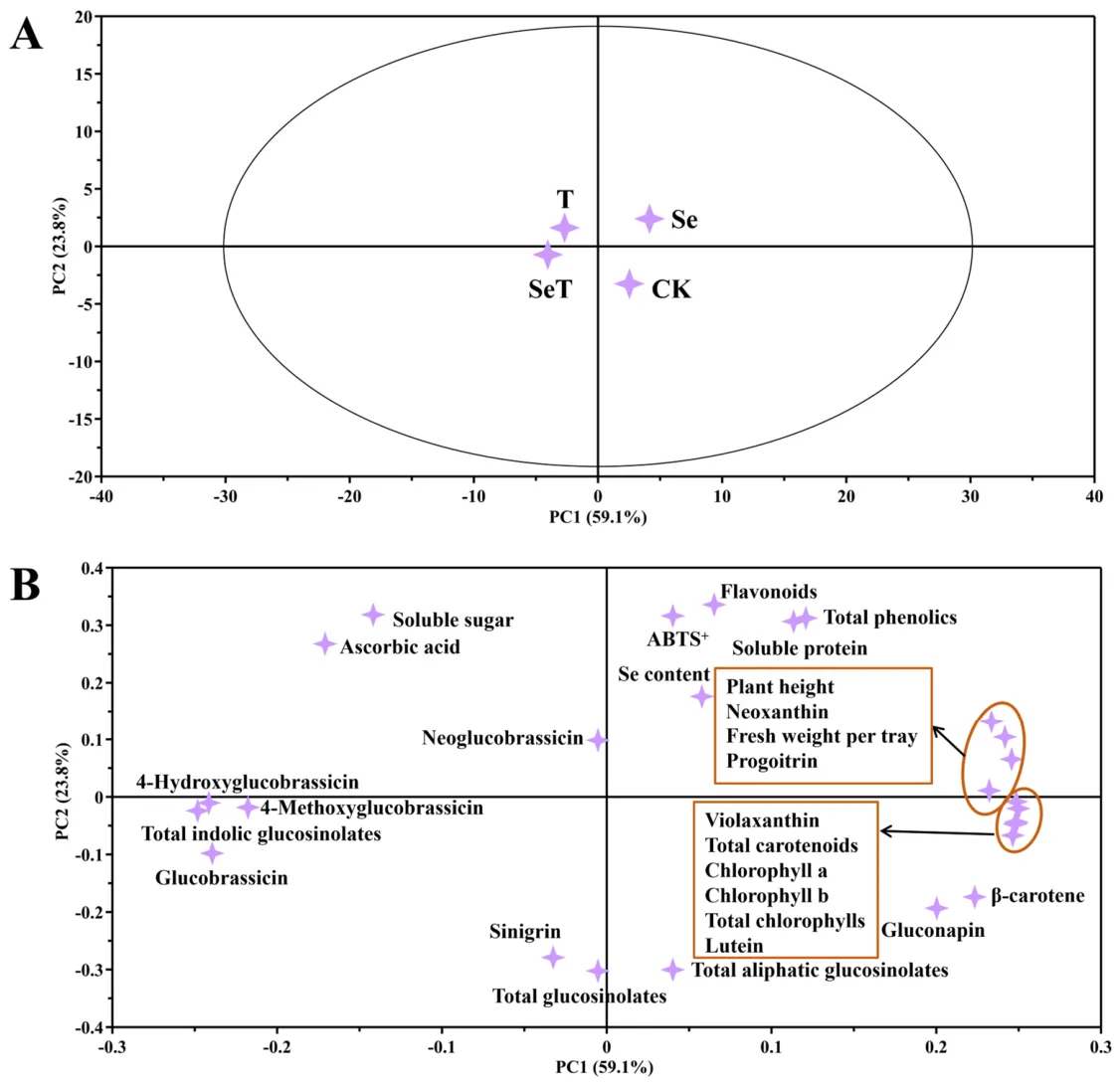
Principal component analysis of mustard sprouts under different treatments. (**A**) PCA score plot; (**B**) loading plot. PCA was performed using four treatment means. This analysis is exploratory and descriptive only. The observed associations cannot be generalized to individual biological replicates and should not be interpreted as evidence of physiological mechanisms. The data matrix used for PCA is provided in Supplementary Table S2. Because some variables are highly overlapping in Figure 5B, orange circles are used to highlight these clustered data points. Arrows point from the circles to orange rectangular boxes, which list the specific variable names corresponding to the data points within each circle.

PCA was used to systematically assess the relationships between the different treatments and the physiological and metabolic traits of mustard sprouts using score and loading plots. PC1 and PC2 explained 59.1% and 23.8% of the total variance, respectively, together accounting for 82.9% of the variation and capturing most of the information in the dataset. The four treatments formed separate trends in the score plot. CK and Se treatments were separated from T and SeT treatments along PC1, indicating that trehalose was the primary factor underlying treatment differentiation. PC2 further distinguished Se-treated from Se-free samples (Figure 5A). The loading plot revealed associations between treatments and growth, antioxidant, and secondary metabolic traits. Se treatment was primarily associated with growth and antioxidant-related traits, including edible fresh weight, plant height, soluble sugar, soluble protein, total chlorophylls, total carotenoids, total phenolics, and flavonoids, whereas SeT treatment was more strongly associated with the accumulation of secondary metabolites, particularly GS (Figure 5B). It should be noted that this PCA was performed using treatment means rather than individual biological replicates; therefore, the separation observed in the score plot is descriptive and cannot be used to infer significant differences among treatments.

### 2.6. Correlation Analysis

**Figure 6:**
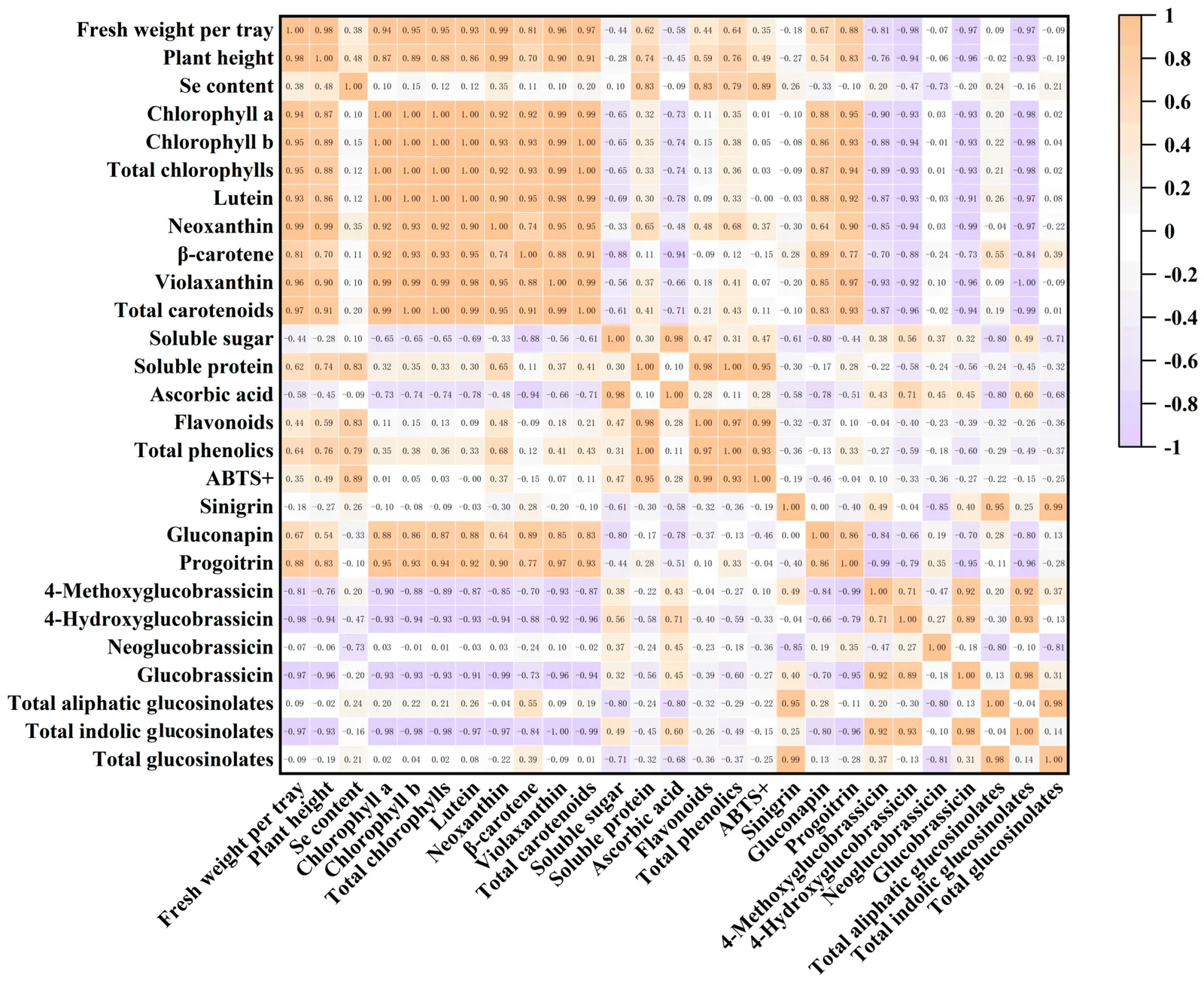
Correlation analysis of mustard sprouts under different treatments. Correlations shown in the figure represent Pearson correlation coefficients above the significance threshold (*p* < 0.05). Correlation analysis was performed using four treatment means. This analysis is exploratory and descriptive only. The observed associations cannot be generalized to individual biological replicates and should not be interpreted as evidence of physiological mechanisms. The raw data matrix used for correlation analysis, together with all correlation coefficients and their corresponding *p* values, is provided in Supplementary Table S2.

Correlation analysis showed that edible fresh weight was positively correlated with plant height (0.98) and total carotenoids content (0.97). Soluble sugar content was positively correlated with ascorbic acid content (0.98). Soluble protein content showed positive correlations with flavonoids (0.98) and total phenolics (1.00). Total glucosinolates content was positively correlated with sinigrin (0.99) and total aliphatic glucosinolates content (0.98). All correlations were statistically significant (*p* < 0.05) (Figure 6). It should be noted that this correlation analysis was performed using treatment means rather than raw observations of independent biological replicates. Accordingly, the observed associations only reflect relationships at the treatment level and cannot be extrapolated to the level of individual biological replicates.

## 3. Discussion

With growing interest in functional foods, cruciferous sprouts, particularly mustard sprouts, have become increasingly popular. Mustard sprouts are rich in various plant-derived nutritional compounds, including GS and phenolic compounds [3]. In recent years, considerable progress has been made in regulating sprouts quality, with studies examining the effects of hormones, plant growth regulators, sugars, and trace elements, providing opportunities for more efficient sprout production. Building on these findings, the present study investigated how single and combined applications of Se and Tre regulate the growth performance and nutritional characteristics of mustard sprouts.

### 3.1. Se Treatment Alone Promoted Growth and Se Enrichment

As an essential micronutrient for plants, Se plays important roles in regulating plant growth and development [21]. Previous studies have demonstrated dose-dependent responses to Se, with appropriate Se levels promoting plant development, whereas excessive Se concentrations exert toxic effects and inhibit growth [22]. In this study, Se treatment significantly enhanced the growth performance of mustard sprouts, as reflected by increased plant height and edible fresh weight. These beneficial effects may be attributed to Se-mediated improvements in photosynthetic capacity and organic substance biosynthesis, which together contribute to increased sprout productivity [8]. Previous studies have similarly reported increased yield and plant height in Se-treated mung bean sprouts, associated with enhanced photosynthesis and the accumulation of photosynthetic products [23]. Appropriate Se concentrations have also been shown to promote growth and biomass accumulation in radish sprouts [7], consistent with our findings. In addition to promoting growth, Se treatment markedly increased the endogenous Se concentration of mustard sprouts, indicating that sodium selenite can serve as an effective Se supplement to enhance both sprout growth and Se accumulation, thereby achieving the dual benefits of increased yield and nutritional enrichment.

### 3.2. Se and Tre Alone and in Combination Improved the Nutritional Quality

As pivotal indicators of vegetable nutritional quality, soluble sugar and soluble protein reflect the intensity of plant carbon and nitrogen metabolism [24]. In this study, all three treatments significantly increased the soluble sugar and soluble protein contents of mustard sprouts. Previous research on radish sprouts similarly showed that exogenous Se markedly promotes the accumulation of soluble sugar and soluble protein [7], while Tre treatment significantly increases soluble sugar content [25]. Se has also been reported to stimulate glucose metabolism, thereby promoting the accumulation of total carbohydrates and crude protein in plants [26]. As an important osmoprotectant, Tre maintains cellular osmotic balance and stabilizes proteins [16]. Soluble protein can also function as osmotic regulators and nutrient reserves, contributing to the synthesis of osmolytes that alleviate osmotic stress [7]. These findings indicate that Se and Tre, individually or in combination, can improve the nutritional quality of sprouts. Specifically, Se and Tre may promote the accumulation of soluble sugar and protein by modulating plant metabolism, thereby contributing to plant protection against stress.

### 3.3. Se and Tre Alone and in Combination Enhanced the Antioxidant Capacity

As an indispensable micronutrient for human health, ascorbic acid acts as an effective ROS scavenger and alleviates oxidative stress-induced damage [27]. Phenolic compounds, including flavonoids and total phenolics, are ubiquitous in plants and play important roles in stress adaptation and antioxidant metabolism; they are also key bioactive components underlying the antioxidant properties of leafy vegetables [28,29]. Multiple studies have shown that exogenous Se and Tre modulate antioxidant defense networks and enhance plant tolerance to diverse biotic and abiotic stresses [30,31]. In this study, Se and Tre, alone or in combination, significantly increased the content of ascorbic acid, flavonoids, and total phenolics, thereby enhancing the antioxidant capacity of mustard sprouts. Se treatment has been reported to promote the accumulation of non-enzymatic antioxidants, enhancing plant resistance to abiotic stress; however, at excessive concentrations, Se may exhibit pro-oxidant effects and impair antioxidant activity [32]. Tre, as a signaling molecule, regulates carbohydrate metabolism through the SnRK1 and ABA pathways, thereby influencing carbon allocation toward antioxidant biosynthesis [18]. In addition, the positive effects of Se on plant antioxidant capacity have been demonstrated in crops such as strawberry [33] and chickpea sprouts [34], while the antioxidant-promoting effects of Tre have been reported in peach [35], litchi [36], and other crops.

### 3.4. Tre Alone and Combined Se + Tre Treatment Promoted the Accumulation of Indolic Glucosinolates

Glucosinolates are unique sulfur- and nitrogen-containing secondary metabolites of cruciferous vegetables with anticancer and chemical defense functions [37]. In this study, Tre treatment alone and in combination with Se significantly promoted the accumulation of total indolic glucosinolates, whereas Se treatment alone significantly reduced their content. First, the inhibitory effect of Se on indolic glucosinolates in mustard sprouts was different from previous studies [11]. Studies have pointed out that Se and sulfur share similar chemical properties, so they can compete during plant uptake and assimilation, potentially interfering with the supply of sulfur-containing precursors required for glucosinolate biosynthesis [13,38]. The decrease of indolic glucosinolates observed in this study is likely due to the limited supply of sulfur-containing precursors caused by Se–sulfur competition. In addition, Se treatment has been reported to reduce indolic glucosinolate levels in Chinese cabbage sprouts, accompanied by downregulation of indolic glucosinolate biosynthesis-related genes [38]. Similarly, in broccoli, Se treatment inhibited the expression of indolic glucosinolate biosynthesis genes, including *CYP79B2* and *CYP83B1* [39]. These findings suggest that the Se-induced reduction in indolic glucosinolates may result from both precursor limitation and transcriptional inhibition, although the underlying mechanisms require further investigation. Notably, it has also been reported to increase the content of indolic glucosinolates, such as indol-3-carbinol in sauerkraut [40]; in a variety of *Cruciferous* sprouts, it was found that the effect of Se biofortification on glucosinolates was species-specific, and the content of glucosinolates in some materials did not decrease or even remained stable [11]. This discrepancy indicates that the effects of Se on glucosinolate metabolism may have positive, negative, or species-dependent effects. Moreover, under Se treatment, antioxidant phenolics and indolic glucosinolates showed the opposite trend, suggesting that this may reflect the redistribution of metabolic resources between secondary metabolism, but it still needs to be further verified [12].

In contrast, Tre appears to promote the accumulation of indolic glucosinolates through multiple mechanisms. Studies have confirmed that Tre, acting as both a signaling molecule and an osmoprotectant, can activate sugar signaling and thereby positively regulate indolic glucosinolate biosynthesis in *Arabidopsis thaliana* [41]. In addition, Tre can enhance the antioxidant defense system and overall stress tolerance of plants. This improved physiological status may provide additional carbon skeletons and energy for the synthesis of energy-intensive secondary metabolites [42]. Under the combined treatment of Se and Tre, the specific contribution of Tre can be understood as a partial buffer for the inhibition of Se-induced indolic glucosinolate accumulation. First, Tre may provide carbon skeleton and energy for glucosinolate biosynthesis through the sugar signaling pathway; second, Tre may reduce Se-induced oxidative damage to the biosynthetic pathway by maintaining the antioxidant defense system. This explanation also clarifies that Tre inhibits plant growth and photosynthetic pigment accumulation while promoting the accumulation of indolic glucosinolates, which reflects the typical growth–defense trade-off phenomenon. It is speculated that Tre may drive plants to redistribute carbon resources through sugar signal-mediated stress response, reduce the input of vegetative growth and photosynthetic pigment synthesis, and preferentially supply carbon sources to defensive secondary metabolic pathways, thereby promoting the accumulation of indole glucosinolates. It should be noted that the above expression is based on the inference of phenotypic association. Whether Se and Tre interact directly at the metabolic level remains to be further explored.

This study systematically investigated the effects of single and combined Se and Tre treatments on the growth and quality of mustard sprouts, but several limitations remain. We focused on phenotypic changes, physiological responses, and the accumulation of related metabolites under exogenous Se and Tre treatments. However, the regulatory effects of these treatments were characterized primarily at the physiological and phenotypic levels, while the underlying mechanisms remain insufficiently resolved. The key genes and molecular regulatory networks involved were not identified, and the core regulatory pathways and mechanisms of action require further investigation. Therefore, future studies should integrate multi-omics approaches and other molecular tools to elucidate how Se and Tre regulate mustard sprout growth and quality, strengthen the theoretical basis of this research, and provide more comprehensive support for the efficient cultivation and quality improvement of mustard sprouts.

## 4. Materials and Methods

### 4.1. Plant Material and Treatments

In this study, the mustard cultivar ‘Chuanbao 18’ was selected as the material. Four experimental treatments were set up in this study, including the control (CK), single selenium treatment (Se), single trehalose treatment (T), and combined selenium and trehalose treatment (SeT). A total of 9 g of mustard seeds was placed in centrifuge tubes and soaked with distilled water or 4 mg·L^−1^ sodium selenite solution at 25 °C for 24 h. After soaking, the seeds were uniformly sown in seedling trays (33 cm × 26 cm × 4.5 cm) for hydroponic cultivation. All trays were incubated in a light incubator with dark culture for the first two days, followed by regular light cultivation. Three biological replicates were set for each treatment, and each seedling tray represented a biological replicate.

The controlled culture parameters were set as light intensity of 80 μmol·m^−2^·s^−1^, diurnal temperature of 25 °C/23 °C (day/night), 16 h light/8 h dark photoperiod, and relative humidity of 75%. The distilled water in seedling trays was renewed every two days, while 6 mmol·L^−1^ trehalose solution was replaced starting from the fifth day of cultivation. On the 10th day after sowing, mustard sprouts were harvested by root cutting, and the fresh weight of aboveground tissues was measured immediately. The samples in each seedling tray were mixed as a biological replicate sample. All collected samples were freeze-dried, ground into fine powder, and stored at −20 °C for subsequent physiological and biochemical determinations. The biochemical determination of each index was carried out independently based on three biologically repeated samples.

### 4.2. Growth Parameters of Sprouts

In total, 30 mustard sprouts were randomly selected from each treatment, with 10 plants per replicate (individual sprouts were regarded as subsamples within each biological replicate), and the plant height of mustard sprouts was measured with a ruler. After sampling, the weight of the aboveground part (the fresh weight of the aboveground part after removing the root) was weighed using a balance and recorded as the fresh weight (the weight of a tray of mustard sprouts) [43].

### 4.3. Se Content

The Se content was determined according to the National Food Safety Standard Determination of Selenium in Food (GB 5009.93-2017) [44], and three biological replicates were set for each treatment.

### 4.4. Chlorophyll and Carotenoid Content

The dry sample was weighed (40 mg), 4 mL of 90% acetone was added to the extract, an ice-bath ultrasound combination was carried out for 20 min, and 4000× *g* centrifugation was conducted at room temperature for 5 min. The supernatant was filtered through a 0.22 μm nylon syringe filter, and HPLC analysis of chlorophylls and carotenoids was performed using an Agilent 1260 instrument and a variable wavelength detector (VWD) (Agilent Technologies, Inc., Palo Alto, CA, USA). The sample (10 μL) was separated on a Waters C18 column (150 × 3.9 mm i.d.; 4 μm particle size) using isopropyl alcohol and 80% acetonitrile–water as the mobile phase at a flow rate of 0.5 mL min^−1^. The column temperature was 30 °C. The mobile phase was isopropanol and 80% acetonitrile–water, the flow rate was 0.5 mL min^−1^, and the column temperature was 30 °C. The absorbance was measured at 448 and 428 nm [45].

### 4.5. Soluble Sugar Content

The steps were as follows: weigh the dry (20 mg) sample in a 10 mL centrifuge tube, add 8 mL of distilled water, vortex for 30 s, carry out extraction for 20 min in a 90 °C water bath, cool, and centrifuge with 4000× *g* for 5 min. The mixture of 1 mL of sample supernatant, 0.5 mL of anthrone-ethyl acetate reagent, and 5 mL concentrated sulfuric acid was quickly reacted at 90 °C in a water bath for 5 min, and the absorbance at 630 nm was measured within 5 min [45].

### 4.6. Soluble Protein Content

The steps were conducted as follows: the dry (20 mg) sample was weighed in a 10 mL centrifuge tube; 8 mL of distilled water was added; the sample was vortexed for 30 s, rested for 40 min, and centrifuged at 4000× *g* for 5 min; 1 mL of supernatant was transferred to a 10 mL centrifuge tube; 5 mL of Coomassie brilliant blue G-250 solution was added; and the absorbance at 595 nm was measured within 20 min [45].

### 4.7. Ascorbic Acid Content

The dry sample (50 mg) was extracted with 5 mL of 1.0% oxalic acid, centrifuged at 4000× *g* for 5 min, filtered with 0.45 μm cellulose acetate filter, and analyzed by HPLC with an Agilent 1260 VWD detector (Agilent Technologies, Inc., Palo Alto, CA, USA). The 10 μL sample was separated on a Waters Spherisorb C18 column (150 × 4.6 mm i.d.; 5 μm particle size) at 30 °C with 0.1% oxalic acid as the solvent at a flow rate of 1.0 mL min^−1^. Ascorbic acid was used as the standard, and the amount of ascorbic acid was calculated from the absorbance value at 243 nm [45].

### 4.8. Flavonoid Content

The dry sample was weighed (40 mg) in a 10 mL centrifuge tube, 3 mL of 50% ethanol was added, the sample was incubated in the dark at room temperature for 24 h, and it was centrifuged at 4000× *g* for 5 min; 1.2 mL of supernatant was mixed with 60 μL of 2% aluminum trichloride and 60 μL of 1 mol L^−1^ potassium acetate, and then 1.68 mL of distilled water was added and left to rest for 40 min. The absorbance was measured at 415 nm [45].

### 4.9. Total Phenolic Content

We weighed the dry (40 mg) sample in a 10 mL centrifuge tube, added 3 mL of 50% ethanol, incubated it in the dark at room temperature for 24 h, and centrifuged it at 4000× *g* for 5 min; 0.3 mL of supernatant was mixed with 1.5 mL of 0.2 mol L^−1^ Folin–Ciocalteu reagent, and 3 min later, 1.2 mL of saturated sodium carbonate was added. After 1 h, the absorbance was measured at 760 nm [45].

### 4.10. ABTS^+^ Assay

ABTS^+^ radicals were generated by the addition of 2.45 mmol L^−1^ ammonium persulfate to a 7 mmol L^−1^ ABTS^+^ solution, and the solution was kept in the dark for 16 h. The ABTS^+^ solution was adjusted with acetate buffer (pH 4.5) to an absorbance of 0.700 (±0.020) at 734 nm. For each extracted sample, a 300 μL solution was added to 3 mL of the ABTS^+^ solution. After 2 h, the absorbance was measured at 734 nm [45].

### 4.11. Glucosinolate Content

The dry (100 mg) sample was weighed in a 10 mL centrifuge tube; 5 mL of distilled water was added, boiled for 10 min, and centrifuged (5 min, 4000× *g*) to collect the supernatant; and the residue was washed with water (5 mL), centrifuged, and merged with the previous extract. The water extract (1 mL) was loaded onto a DEAE-Sephadex A-25 column. The glucosinolates were converted into their desulpho analogues by overnight treatment with 100 μL of 0.1% aryl sulfatase, and the desulphoglucosinolates were eluted with 1 mL of water. HPLC analysis of glucosinolates was performed using an Agilent 1260 HPLC instrument equipped with a VWD detector (Agilent Technologies, Inc., Palo Alto, CA, USA). At 30 °C, on a Waters Spherisorb C18 column (250 × 4.6 mm i.d.; 5 μm particle size), acetonitrile and water were used to separate the samples at a flow rate of 1.0 mL min^−1^. The absorbance was detected at 226 nm. Glucosinolates were quantified by using *ortho*-Nitrophenyl β-D-galactopyranoside as an internal standard and considering the response factor of each glucosinolate [45].

### 4.12. Statistical Analysis

Statistical two-way ANOVA analysis was completed on DPS 9.01, and Duncan’s multiple-range test was adopted to distinguish significant differences when *p* < 0.05. Principal component analysis (PCA) modeling was processed through SIMCA 14.1 (Umetrics AB, Malmö, Sweden); all variables were mean-centered and scaled to unit variance (UV scaling, autoscale) before multivariate analysis. Correlation analysis heat maps were generated by OriginPro 2024 (Origin Lab Corporation, Northampton, MA, USA). The significance analysis in this study was based on three biologically replicated sample data, and principal component analysis (PCA) and correlation analysis were performed using the mean of each treatment group. In the statistical analysis of plant height, the average value of all subsamples in each biological replicate was calculated first, and then the average value of each biological replicate was used for significance analysis.

## 5. Conclusions

This study demonstrated that Se and Tre can effectively enhance the nutritional quality of mustard sprouts. Se treatment alone significantly promoted sprout growth, increasing edible fresh eight and plant height. Se and Tre alone and their combined treatment all increased the content of soluble sugar, soluble protein, ascorbic acid, flavonoids, and total phenolics, thereby promoting nutrient accumulation and enhancing antioxidant capacity. In addition, Tre alone and the combined treatment significantly increased the accumulation of total indolic glucosinolates, whereas Se alone significantly decreased their content, and the combined treatment was especially advantageous in the accumulation of indolic glucosinolates. Overall, Se and Tre exhibited differential effects on the growth, nutritional quality, and secondary metabolism of mustard sprouts, indicating a trade-off between these treatments. Notably, Se alone exerted relatively beneficial effects on the nutritional quality and antioxidant capacity of mustard sprouts, while the application of Tre alone and the combined treatment promoted the accumulation of secondary metabolites, accompanied by the inhibition of plant growth and the decrease in photosynthetic pigment content.

## Data Availability

The original contributions presented in the study are included in the article; further inquiries can be directed to the corresponding author.

## Supplementary Materials

The following supporting information can be downloaded at: https://www.mdpi.com/article/10.3390/plants15213371/s1, Table S1: Results of two-way analysis of variance for each response variable (fixed model); Table S2: Correlation and principal component analysis (PCA) data matrix and correlation analysis of the correlation coefficient and *p*-value.
